# Serotonin Receptor 2A/C Is Involved in Electroacupuncture Inhibition of Pain in an Osteoarthritis Rat Model

**DOI:** 10.1093/ecam/neq016

**Published:** 2011-01-09

**Authors:** Aihui Li, Yu Zhang, Lixing Lao, Jiajia Xin, Ke Ren, Brian M. Berman, Rui-Xin Zhang

**Affiliations:** ^1^Center for Integrative Medicine, School of Medicine, University of Maryland, Baltimore, MD 21201, USA; ^2^Department of Neurobiology, Shanxi Medical University, Taiyuan 030001, Shanxi, PR, China; ^3^Department of Neural and Pain Sciences, Dental School, University of Maryland, Baltimore, MD 21201, USA

## Abstract

Osteoarthritis currently has no cure. Acupuncture can benefit patients with knee osteoarthritis by providing pain relief, improving joint function and serving as an effective complement to standard care. However, the underlying mechanisms of its effects are still not completely understood. The present study, an investigation of the effectiveness and mechanisms of electroacupuncture (EA) in attenuating osteoarthritis pain in a rat model, is focused on the involvement of 5-hydroxytryptamine 2A/C (5-HT2A/C) receptors, which play an important role in pain modulation at the spinal level. Osteoarthritis was induced under isoflurane anesthesia by a single intraarticular injection of monosodium iodoacetate (3 mg/50 *μ*L/rat) into one hind leg of each rat. EA was given at acupoints GB 30 and ST 36 on days 1–4 after the injection. Vehicle or ketanserin, a 5-HT2A/C receptor antagonist, was given intraperitoneally (1 mg kg^−1^) or intrathecally (5 *μ*g or 10 *μ*g/10 *μ*L), 30 min before each EA treatment. Assessment of weight-bearing difference between injected and uninjected hind legs was done on days 0, 1–4 and 7. Fos /serotonin and serotonin/Fluorogold double labeling were performed to determine EA activation of serotonergic neurons in the nucleus raphe magnus (NRM) that project to spinal cord. The results showed that EA significantly decreases weight-bearing difference compared to sham EA. Ketanserin pretreatment blocked the analgesic effect of EA but did not influence weight bearing in sham EA control rats. EA also activated serotonergic NRM neurons that project to the spinal cord. These data show that EA inhibits osteoarthritis-induced pain by enhancing spinal 5-HT2A/2C receptor activity.

## 1. Introduction

Osteoarthritis, a widespread condition, affects nearly 21 million people in the United States. Its prevalence after age 65 is about 60% in men and 70% in women [1]. Its main symptom is chronic pain that causes loss of mobility and often stiffness.

Osteoarthritis is irreversible, and currently there is no cure. The purpose of pharmacological treatments is to control pain and reduce functional limitation. Frequently used medications such as non-steroidal anti-inflammatory drugs have serious and even dangerous side effects, including bleeding and perforated ulcers [2, 3]. Cyclooxygenase-2 inhibitors can reduce the gastrointestinal side effects, but these carry an elevated risk for cardiovascular disease, and some have now been withdrawn from the market [4]. Thus, more and more patients turn to complementary and alternative therapies.

Acupuncture, a commonly used complementary therapy with few or no adverse effects, has been used in China for thousands of years to treat a variety of conditions, including pain. Its popularity in western countries has been increasing in recent years. There are reports that acupuncture can benefit patients with knee osteoarthritis by providing pain relief, improving joint function and serving as an effective complement to standard care [5–8]. However, its underlying mechanisms are still not completely understood.

In the central nervous system, there are several 5-HT receptor subtypes, of which both 5-HT2A and 5-HT2C are important in the perception of pain. It has been reported that m-5-HT, a 5-HT2A/2C receptor agonist, dosage-dependently suppressed the number of flinches in the formalin test and attenuated thermal hyperalgesia in a chronic constriction injury model. These effects were reversed by a 5-HT2A/2C antagonist, ketanserin, which blocks the 5-HT2A and 5-HT2C receptors [9]. The data demonstrate that these receptors play a key role in persistent pain.

The aims of the present study were to test whether electroacupuncture (EA) effectively attenuates pain in an osteoarthritis rat model and whether 5-HT2A/C receptors are involved in such attenuation. Serotonin, an important neurotransmitter, modulates the transmission of noxious messages at the spinal level [10]. Intrathecally (i.t.) administered, it produces anti-nociception in rats, rabbits and cats [11]. Since serotonin neurons in the NRM participate in pain modulation [10], we also double labeled serotonin and Fluorogold, a retrograde tracer, which was injected into the spinal cord, and serotonin and Fos, an indicator of neuron activation, to determine whether EA activates serotonergic NRM neurons that project to the spinal cord.

## 2. Methods

### 2.1. Animal Preparation

Male Sprague-Dawley rats weighing 280–350 g (Harlan, Indianapolis, IN) were kept under controlled environmental conditions (22°C, relative humidity 40%–60%, 12-h alternate light-dark cycles, food and water *ad libitum*). The animal protocols were approved by the Institutional Animal Care and Use Committee of the University of Maryland School of Medicine.

### 2.2. Experimental Design

Osteoarthritis was induced in all rats by a single intra-articular injection of monosodium iodoacetate (Sigma) dissolved in saline [12]. Briefly, rats were anesthetized with isoflurane, and monosodium iodoacetate (3 mg/50 *μ*L/rat) was injected into the left knee joint cavity using a 29-gauge needle inserted through the patellar tendon. To set up the osteoarthritis rat model in our lab, two groups of rats (*n* = 8 per group) were used in the first experiment: one received monosodium iodoacetate and the other received saline. Hind limb weight-bearing differences were assessed on days 0, 1–4 and 7 after the intra-articular injection. To investigate EA inhibition of pain in the arthritis rat model and the participation of 5HT2A/2C receptors in this effect, the monosodium iodoacetate-injected rats received intraperitoneal (i.p.) ketanserin or vehicle and were randomly divided into four groups (*n* = 8 per group): EA plus ketanserin (1 mg kg^−1^, i.p.) dissolved in 10% DMSO, EA plus vehicle (10% DMSO in saline), sham EA plus ketanserin and sham EA plus vehicle. To specify whether an 5HT2A/2C antagonist acted in the spinal cord, another set of monosodium iodoacetate-injected rats received ketanserin or vehicle and were randomly divided into EA plus ketanserin (10 *μ*g/10 *μ*L, i.t.), EA plus ketanserin (5 *μ*g/10 *μ*L, i.t.) or EA plus vehicle (i.t.) groups. EA was given on days 1–4 30 min after the drug or vehicle injection. Behavioral tests to assess pain were given on day 0 for baseline, day 1 before EA treatment to confirm the development of arthritis pain, days 2–4 17 h post-EA to determine the effect of EA on arthritis pain and day 7 to observe long-term effects. It should be noted that we performed behavioral tests on the day after EA treatment to rule out any effect from the isoflurane.

Another six rats were used in an immunofluorescence study. Three were treated once with EA and perfused 2 h later. Their brainstems were removed and double labeled for Fos and serotonin. The spinal cords of other three were injected with Fluorogold, and Fluorogold and serotonin were double labeled in the NRM.

### 2.3. Pain Assessment

Pain-related behaviors were assessed using a hind limb weight-bearing apparatus (Model-600, IITC Life Science). Rats were allowed to acclimate to the testing apparatus for 30 min a day, 2-3 days before experiment. The amount of weight supported by each hind leg was measured automatically and separately every 5 s. At each time point, five readings were taken for each rat and averaged first for each individual and then for the group. Weight-bearing difference between the two legs was presented as the percentage of weight borne by the ipsilateral leg and was determined using the following formula: % weight on the ipsilateral leg = weight on the ipsilateral leg/ (weight on the contralateral leg + weight on the ipsilateral leg) ×100%.

### 2.4. Acupuncture Treatment Procedures

A 30-min EA treatment was given once a day on days 1–4 after the iodoacetate injection. EA parameters of 10 Hz at 2 mA and 0.4 ms pulse width, which showed significant antihyperalgesic effects on a bone cancer pain rat model in our previous studies [13], were used. The equivalents of human acupoints Huantiao (GB30) and Zusanli (ST 36) were chosen for bilateral needling based on traditional Chinese medicine meridian theory [14]. In humans, GB30 is located at the junction of the lateral 1/3 and medial 2/3 of the distance between the greater trochanter and the hiatus of the sacrum; underneath are the sciatic nerve, inferior gluteal nerve and gluteal muscles [15]. ST 36 is located on the anterior aspect of the lower leg, 3 U (based on the standard acupuncture measurement of 16 U between the knee and the ankle joint) below the knee joint and one finger-breadth (middle finger) lateral to the anterior crest of the tibia. The anatomical structure of this point involves the tibialis anterior muscle; underneath is the deep peroneal nerve. These acupoints were located on the rat's hind limbs using the comparable anatomical landmarks.

The animals were gently handled for 30 min each day for 2-3 days and habituated to the handling and experimental environment. After cleaning with alcohol swabs and anesthetizing with isoflurane using a Vaporizer (Harvard Apparatus), two disposable acupuncture needles (gauge #32, 0.5 inch in length) were inserted *∼*0.5 inch into GB 30 and 0.25 inch into ST 36 on the animal's hind limbs. On each leg, a pair of electrodes was attached to the ends of the needles. EA stimulation was delivered by a stimulator (Electrostimulator 8-C, Pantheon Research Inc.) at 10 Hz, 2 mA and 0.4 ms pulse width for 30 min on days 1–4. While frequency was held constant, intensity was adjusted slowly to the designated level of 2 mA. Mild muscle twitching was observed. A symmetrical biphasic wave was delivered to the electrodes on each leg so that the electrode was alternately positive and negative, and GB 30 and ST 36 were stimulated alternately. Because the rats were anesthetized during acupuncture treatment, they were not restrained.

For sham treatment control, acupuncture needles were inserted bilaterally into GB 30 and ST 36 without electrical stimulation or manual manipulation. This procedure is comparable to actual treatment but produces little therapeutic effect: our previous study showed that needles inserted into active acupoints, but given no electrical or manual stimulation, do not produce analgesia [16].

### 2.5. Intrathecal Drug Delivery

Acute lumbar punctures were performed as previously described [17]. A PE10 polyethylene tube (Clay Adams) was submerged in 70°C water, stretched to about 150% of the original length to reduce its diameter and used as an injection catheter. With a 29-gauge needle, another 10-cm PE10 tube was connected to one end of the catheter and then to a 50-*μ*L glass Hamilton syringe with a PE50 tube. The injection catheter was pre-filled with 10 *μ*L of drug or vehicle and 5 *μ*L of saline separated by a small air bubble. Under isoflurane anesthesia, the dorsal pelvic area was shaved and swabbed with 70% alcohol. A 21-gauge sterile needle with the plastic hub removed was inserted between lumbar vertebrae L5 and L6. The catheter was inserted into the guide needle and rostrally advanced 4 cm from the tip of the needle into the lumber enlargement, where its arrival was confirmed by a tail-flick. The drug, or vehicle, was injected and followed by a saline flush. 5 min after injection, the catheter was withdrawn and the needle was removed from the intervertebral space.

### 2.6. Immunofluorescence

Rats were deeply anesthetized with sodium pentobarbital (80 mg kg^−1^, i.p.) and perfused transcardially with 100 mL of saline followed by 500 mL of 4% paraformaldehyde in 0.1 M phosphate buffer at pH 7.4. The brain stem was removed, immersed in the same fixative for 2 h and transferred to a solution of 30% sucrose in a phosphate buffer for 72 h. 40-*μ*m-thick sections were cut with a cryostat at –20°C. The sections were processed with a free-floating stain method. For double immunofluorescence labeling, brain stem sections were blocked in PBS with 10% normal donkey serum for 60 min and incubated overnight at room temperature in a mixture of rabbit anti-Fos (Oncogene, 1 : 1000) and goat anti-serotonin (ImmunoStar, 1 : 250). After three 10-min washings in PBS, sections were incubated in a mixture of CY2-conjugated donkey anti-rabbit (1 : 50, Jackson ImmunoResearch Laboratories) and CY3-conjugated donkey anti-goat (1 : 200) for 1 h at room temperature. Control sections were similarly processed, except that the primary antisera were omitted. The stained sections were mounted on gelatin-coated slides, coverslipped with aqueous mounting medium (Biomeda Corp., CA, USA) and examined under a Nikon fluorescence microscope. Control sections without primary antiserum showed no immunoreactive staining.

For serotonin and Fluorogold double labeling, Fluorogold (4%, 0.2 *μ*L) was injected into the lumbar spinal cord after a laminectomy under anesthesia with pentobarbital sodium (50 mg kg^−1^, i.p.). Four days later, the rats were perfused as above. The brain stem sections were incubated in a mixture of rabbit anti-Fluorogold (Chemicon, 1 : 1000) and goat anti-serotonin (ImmunoStar, 1 : 250). After three 10-min washings in PBS, sections were incubated in a mixture of CY2-conjugated donkey anti-rabbit (1 : 50) and CY3-conjugated donkey anti-goat (1 : 200) for 1 h at room temperature and then treated as above.

### 2.7. Data Analysis

Hind limb weight-bearing differences were presented as percentage changes and analyzed using analysis of variance with repeated measures followed by the *post hoc* Multiple Comparisons Test (Statistical Analysis System). *P* < .05 was set as the level of statistical significance.

## 3. Results

### 3.1. An Intra-Articular Injection of Iodoacetate Decreased Weight-Bearing Ability

**Figure 1:**
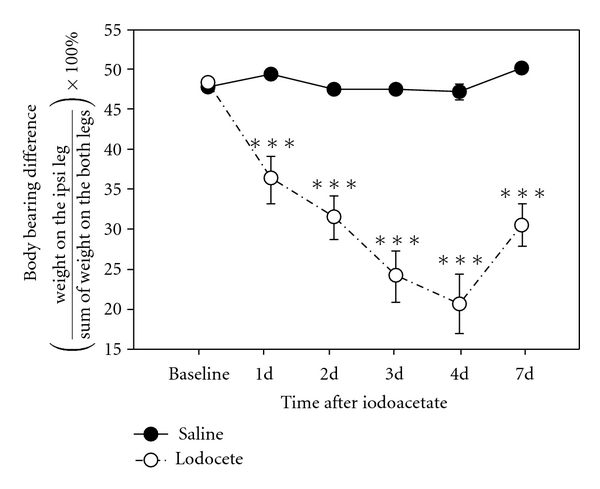
Effect of a unilateral intra-articular injection of monosodium iodoacetate (3 mg/50 *μ*L/rat) on the weight-bearing ability of hind legs (*n* = 8). Note that iodoacetate-injected rats bore significantly less weight on the ipsilateral hind limb than did saline-injected rats. Abscissas: the time points from the 1st to the 7th day after iodoacetate; ordinate: body weight difference. ***P < .001 versus saline.

Before iodoacetate was injected into the knee joint cavity, there was no significant difference between right and left hind limb weight bearing in the groups. After an iodoacetate injection, the ipsilateral hind limb bore significantly (*P* < .001) less weight than did saline injected legs (Figure 1), suggesting that the iodoacetate induced ipsilateral pain.

### 3.2. Systemic Ketanserin Prevented EA Restoration of Weight-Bearing Ability in the Iodoacetate-Injected Knee

**Figure 2:**
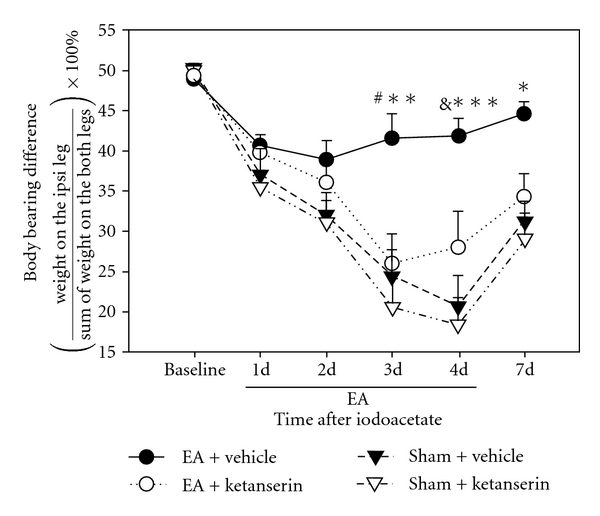
Effect of EA and a systemic injection of ketanserin on weight bearing in rats with osteoarthritis. EA significantly decreased weight-bearing differences compared to sham EA on days 3–7 after iodoacetate. Ketanserin pretreatment blocked the analgesic effect of EA but did not influence weight bearing in sham EA control rats. *P < .05, **P < .01, ***P < .001 versus sham + vehicle; ^#^ *P* < .01, ^&^ *P* < .05 versus EA + ketanserin.

As shown in Figure 2, EA treatment (EA + vehicle) significantly restored the weight-bearing ability of the ipsilateral leg on days 3 (41.6 ± 2.9 versus 24.3 ± 3.4, *P* < .01), 4 (41.8 ± 2.1 versus 20.6 ± 3.7, *P* < .001) and 7 (44.6 ± 1.5 versus 30.2 ± 2.8, *P* < .05) after the iodoacetate injection compared to sham EA control (sham EA + vehicle). This indicates that EA inhibited the iodoacetate-induced pain. Comparison between EA + ketanserin (i.p.) and EA + vehicle (i.p.) indicates that ketanserin blocked EA inhibition of the iodoacetate-induced pain on days 3 (25.84 ± 3.7 versus 41.6 ± 2.9, *P* < .01) and 4 (28.0 ± 4.3 versus 41.8 ± 2.1, *P* < .05). Ketanserin treatment plus sham EA produced the same weight-bearing difference as that produced by vehicle plus sham EA, suggesting that ketanserin alone did not influence the pain.

### 3.3. Ketanserin Blocked EA Alleviation of the Weight-Bearing Decrease

**Figure 3:**
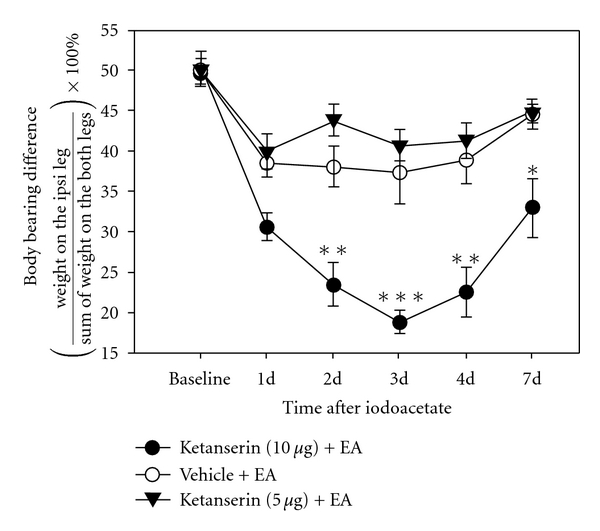
Effect of an i.t. ketanserin injection on EA analgesia. Ketanserin at 10 *μ*g pretreatment significantly blocked the analgesic effect of EA on days 2–7 after iodoacetate. There is no significant difference between the 5 *μ*g and vehicle groups. *P < .05, **P < .01, ***P < .001 versus vehicle control.

As shown in Figure 3, rats given EA plus 10 *μ*g of i.t. ketanserin put significantly less weight on the ipsilateral leg than did rats with EA plus vehicle on days 2 (23.5 ± 2.7 versus 38.1 ± 2.5, *P* < .01), 3 (18.9 ± 1.4 versus 37.3 ± 3.9 versus, *P* < .001), 4 (22.5 ± 3.1 versus 38.8 ± 2.8, *P* < .01) and 7 (32.9 ± 3.6 versus 44.6 ± 1.8, *P* < .05). Weight bearing was not significantly different between rats given EA plus 5 *μ*g of ketanserin and those given EA plus vehicle, although the former showed a slightly greater ability to bear weight. This indicates that EA may inhibit pain by inducing serotonin release in the spinal cord.

### 3.4. EA-Activated Serotonin-Containing NRM Neurons that Project to the Spinal Cord

**Figure 4:**
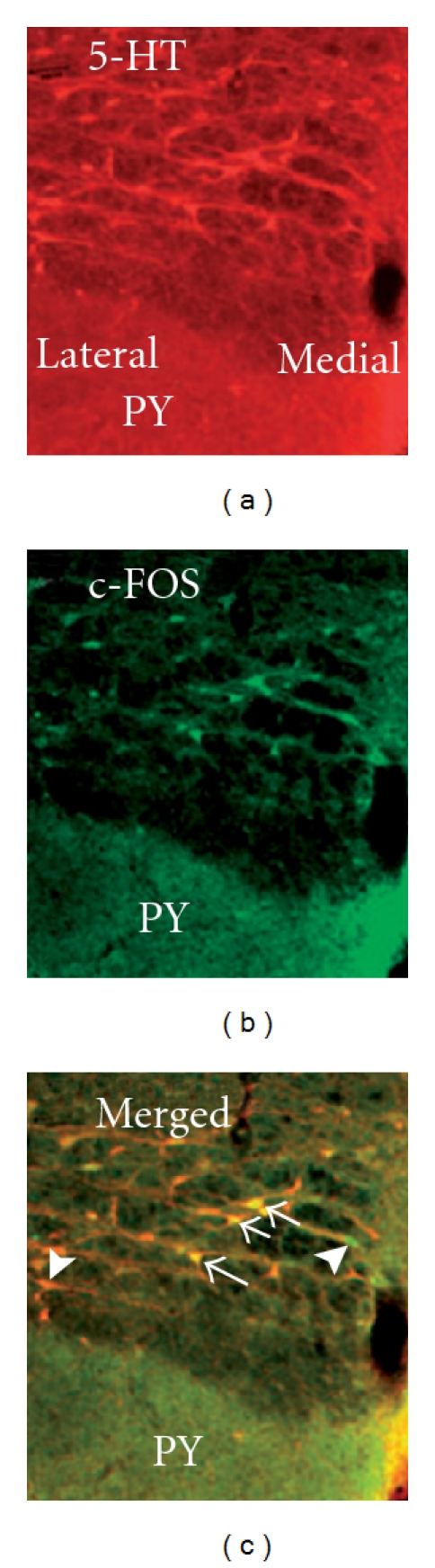
Co-localization of Fos and 5-HT in the nucleus raphe magnus. (a) The 5-HT-immunoreactive neurons; (b) Fos-immunoreactive profiles; (c) Merged graphs of (a) and (b). Arrows indicate double-labeled 5-HT/Fos neurons (yellow). Arrow heads point to single-labeled neurons.

**Figure 5:**
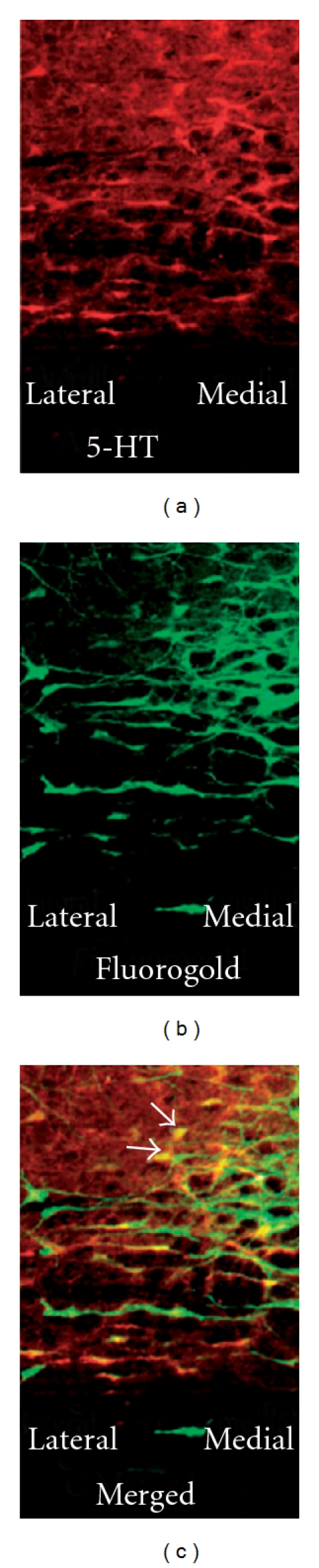
Representative photograph showing the co-localization of serotonin and Fluorogold in the nucleus raphe magnus. (a) The 5-HT-immunoreactive neurons; (b) Fluorogold-labeled neurons projecting to the spinal cord; (c) Merged graphs of (a) and (b). Arrows indicate double-labeled 5-HT/Fluorogold neurons (yellow).

Immunofluorescence double staining of serotonin and Fos demonstrated that EA induced Fos expression in the NRM (Figure 4(b)). Most EA-induced Fos was co-localized with serotonin (Figure 4(c)), which indicates that EA activated serotonergic neurons in this nucleus. Double staining of serotonin and the retrograde tracer Fluorogold, which was injected into the lumbar spinal cord, demonstrated that about 50% of NRM neurons that project to the spinal cord contained serotonin (Figures 5(a)–5(c)).

## 4. Discussion

In the present study, EA treatment significantly decreased the weight-bearing difference between healthy and inoculated hind legs when compared to sham EA in the iodoacetate-induced osteoarthritis rat model. This suggests that EA treatment significantly relieves spontaneous pain in this model. The data are consistent with a previous report that EA significantly improved weight-bearing force in an animal model of arthritic pain induced by injecting carrageenan into the knee joint cavity [18]. Clinical trials have also reported that, compared to control, EA significantly improved pain and joint function as assessed with the Western Ontario and McMaster Universities' Osteoarthritis Index in patients with knee osteoarthritis during a long-term follow-up period of 8–26 weeks [6, 7]. These basic and clinical studies demonstrate that acupuncture can benefit osteoarthritis patients.

Moreover, our study showed that ketanserin, a selective 5-HT2A/C receptor antagonist, produced no significant effect in sham EA control osteoarthritis rats. This suggests that the sham EA did not activate the serotonin system. However, the analgesic effect of EA on the osteoarthritis rat model was blocked by systemic ketanserin (Figure 2), indicating that EA treatment induced the release of serotonin, which activated 5-HT2A/2C receptors to alleviate pain. Further, i.t. ketanserin at 10 *μ*g, but not 5 *μ*g, significantly blocked the effect of EA, demonstrating that the ketanserin dosage-dependently neutralizes the EA effect by blocking the 5-HT2A/2C receptors. Previous study shows that the spinal cord contains abundant 5-HT 2A and 2C receptors [19, 20]. Our data suggest that EA treatment induces the release of serotonin, which activates 5-HT2A/2C receptors in the spinal cord to inhibit pain and increase weight-bearing ability in the limb ipsilateral to the injury. This is consistent with previous studies which showed that i.t. administration of m-5-HT, a 5-HT2A/2C receptor agonist, dosage-dependently suppressed the number of flinches in the formalin test and attenuated thermal hyperalgesia in a rat model of neuropathic pain [9]. Our data are also in accord with a previous report that ketanserin at 10 *μ*g attenuated the 5-HT-produced anti-nociceptive effect [21].

In contrast to our study, a previous study demonstrates that spinal 5-HT1A and 5-HT3 receptor antagonists, but not the HT2A/2C receptor antagonist ketanserin, blocked EA-produced inhibition of cold allodynia in a rat model of neuropathic pain [22]. These data suggest that EA may involve different or multiple mechanisms according to the nature of the noxious stimulus (e.g., cold allodynia versus spontaneous pain). However, the involvement of 5-HT1A and 5-HT3 receptors in EA inhibition of arthritis-induced pain warrants further study, since an i.t. injection of the 5-HT3 receptor agonist induced significant anti-nociceptive effects assessed with paw pressure test [21], and an i.t. injection of the selective 5-HT1A receptor antagonist blocked the anti-nociceptive action of 5-HT in formalin-induced pain [23]. Another study demonstrates that EA may activate serotonin neurons in the nucleus raphe pallidus to modulate cardiovascular function [24]. In our study, EA activated serotonergic neurons in the NRM, which sends serotonergic descending fibers to the spinal cord to modulate pain. Collectively, these studies suggest that EA activates different areas in the brain that, in turn, modulate various functions.

**Figure 6:**
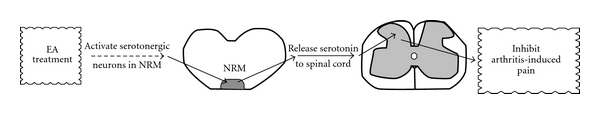
A schematic illustration of the mechanisms of EA inhibition of pain.

In summary, this study shows that EA can be used to activate serotoninergic NRM neurons that project to the spinal cord, induce spinal serotonin release and stimulate 5-HT2A/2c receptor activities at the spinal cord to inhibit osteoarthritis-induced pain (Figure 6).

## Funding

National Institutes of Health grant P01 AT002605.

## References

[1] Sarzi-Puttini P, Cimmino MA, Scarpa R (2005). Osteoarthritis: an overview of the disease and its treatment strategies. *Seminars in Arthritis and Rheumatism*.

[2] Green GA (2001). Understanding NSAIDs: from aspirin to COX-2. *Clinical Cornerstone*.

[3] Gutthann SP, Garcia Rodriguez LA, Raiford DS (1997). Individual nonsteroidal antiinflammatory drugs and other risk factors for upper gastrointestinal bleeding and perforation. *Epidemiology*.

[4] Juni P, Reichenbach S, Egger M (2005). COX 2 inhibitors, traditional NSAIDs, and the heart: adverse event data from clinical trials must inform decision making. *British Medical Journal*.

[5] Liu T, Liu C (2006). Acupuncture for treating osteoarthritis of the knee and the hip. *Arthritis and Rheumatism*.

[6] Berman BM, Lao L, Langenberg P, Lee WL, Gilpin AMK, Hochberg MC (2004). Effectiveness of acupuncture as adjunctive therapy in osteoarthritis of the knee. A randomized, controlled trial. *Annals of Internal Medicine*.

[7] Witt C, Brinkhaus B, Jena S (2005). Acupuncture in patients with osteoarthritis of the knee: a randomised trial. *The Lancet*.

[8] Miller E, Maimon Y, Rosenblatt Y Delayed effect of acupuncture treatment in OA of the knee: a blinded, randomized, controlled trial.

[9] Sasaki M, Obata H, Saito S, Goto F (2003). Antinociception with intrathecal *α*-methyl-5-hydroxytryptamine, a 5-hydroxytryptamine2A/2C receptor agonist, in two rat models of sustained pain. *Anesthesia and Analgesia*.

[10] Millan MJ (2002). Descending control of pain. *Progress in Neurobiology*.

[11] Yaksh TL, Wilson PR (1979). Spinal serotonin terminal system mediates antinociception. *Journal of Pharmacology and Experimental Therapeutics*.

[12] Pomonis JD, Boulet JM, Gottshall SL (2005). Development and pharmacological characterization of a rat model of osteoarthritis pain. *Pain*.

[13] Zhang R-X, Li A, Liu B (2007). Electroacupuncture attenuates bone cancer pain and inhibits spinal interleukin-1[*β*] expression in a rat model. *Anesthesia and Analgesia*.

[14] O’Connor J, Bensky D (1981). *Acupuncture: A Comprehensive Text*.

[15] Cheng X (1999). *Chinese Acupuncture and Moxibustion*.

[16] Lao L, Zhang R-X, Zhang G, Wang X, Berman BM, Ren K (2004). A parametric study of electroacupuncture on persistent hyperalgesia and Fos protein expression in rats. *Brain Research*.

[17] Milligan ED, Sloane EM, Langer SJ (2005). Controlling neurophatic pain by adeno-associated virus driven production of the anti-inflammatory cytokine, interleukin-10. *Molecular Pain*.

[18] Oh JH, Bai SJ, Cho Z-H (2006). Pain-relieving effects of acupuncture and electroacupuncture in an animal model of arthritic pain. *International Journal of Neuroscience*.

[19] Doly S, Madeira A, Fischer J (2004). The 5-HT2A receptor is widely distributed in the rat spinal cord and mainly localized at the plasma membrane of postsynaptic neurons. *Journal of Comparative Neurology*.

[20] Fonseca MI, Ni YG, Dunning DD, Miledi R (2001). Distribution of serotonin 2A, 2C and 3 receptor mRNA in spinal cord and medulla oblongata. *Molecular Brain Research*.

[21] Bardin L, Lavarenne J, Eschalier A (2000). Serotonin receptor subtypes involved in the spinal antinociceptive effect of 5-HT in rats. *Pain*.

[22] Sun KK, Jung HP, Sang JB (2005). Effects of electroacupuncture on cold allodynia in a rat model of neuropathic pain: mediation by spinal adrenergic and serotonergic receptors. *Experimental Neurology*.

[23] Bonnefont J, Chapuy E, Clottes E, Alloui A, Eschalier A (2005). Spinal 5-HT1A receptors differentially influence nociceptive processing according to the nature of the noxious stimulus in rats: effect of WAY-100635 on the antinociceptive activities of paracetamol, venlafaxine and 5-HT. *Pain*.

[24] Guo Z-L, Moazzami AR, Tjen-A-Looi S, Longhurst JC (2008). Responses of opioid and serotonin containing medullary raphe neurons to electroacupuncture. *Brain Research*.

